# Therapeutic monitoring of mood stabilizers in bipolar disorder

**DOI:** 10.1192/j.eurpsy.2021.1296

**Published:** 2021-08-13

**Authors:** D. Falfel, H. Ben Ammar, G. Hamdi, E. Khelifa, L. Mnif

**Affiliations:** Psychiatry Department, Razi Hospital, Manouba, Tunisia

**Keywords:** bipolar disorder, lithium, Mood stabilizer, therapeutic monitoring

## Abstract

**Introduction:**

Efficacy of lithium is well documented in the literature, making it the gold standard treatment. However, its use declines with the advent of anticonvulsants. This raises the question about monitoring of mood stabilizers in practice.

**Objectives:**

The aims of this study were to determine the prophylactic lithium response in patients followed for bipolar disorder and compared to those of anticonvulsants and assess the mood stabilizers monitoring procedures in clinical practice.

**Methods:**

A retrospective study was conducted, over a period of six months, with patients followed for bipolar disorder stabilized under the same mood stabilizer (lithium or anticonvulsant) for at least one year. The participants were divided into two groups according to the mood stabilizing treatment. The two groups were compared according to socio-demographic, clinical and evolutionary profiles as well as the prophylactic response to treatment.

**Results:**

Patients included were 64 in the study, 28 received lithium and 36 received anticonvulsants. The socio-demographic profile and clinical characteristics were similar in two groups, except for the average total number of mood episodes. Retrospective evaluation of the prophylactic response by ALDA scale showed a significantly higher mean total score in patients receiving lithium (5.9 ± 2.8 versus 2.58 ± 2.4, p = 0.025).). Ten of them were in compliance with the recommendations; while 19.44% received anticonvulsants had all the monitoring parameters within the recommended time frame.

**Conclusions:**

Thymoregulators significantly modify the disease’s prognosis. Practitioners will attach particular special attention to distinguish the therapeutic efficacy of the side effects which are numerous and sometimes serious.

